# Supplementary material for: A Critical View of the Application of the APEX Software (Aqueous Photochemistry of Environmentally-Occurring Xenobiotics) to Predict Photoreaction Kinetics in Surface Freshwaters
Source: Molecules. 2019 Dec 18;25(1):9. doi: 10.3390/molecules25010009 (PMC7017383; doi:10.3390/molecules25010009)
Supplement: Supplementary file 1 [file molecules-25-00009-s001.zip › 3.2.4_gcc-4.4.0.zip!/3.2.4_gcc-4.4.0/3.2.4_gcc-4.4.0/doc/octave/html/interpreter/Audio-Processing.html]

Audio Processing - Untitled


Next: Object Oriented Programming,
Previous: Image Processing,
Up: Top


---

## 32 Audio Processing

Octave provides a few functions for dealing with audio data. An audio
`sample' is a single output value from an A/D converter, i.e., a small
integer number (usually 8 or 16 bits), and audio data is just a series
of such samples. It can be characterized by three parameters: the
sampling rate (measured in samples per second or Hz, e.g., 8000 or
44100), the number of bits per sample (e.g., 8 or 16), and the number of
channels (1 for mono, 2 for stereo, etc.).

There are many different formats for representing such data. Currently,
only the two most popular, *linear encoding* and *mu-law
encoding*, are supported by Octave. There is an excellent FAQ on audio
formats by Guido van Rossum <guido@cwi.nl> which can be found at any
FAQ ftp site, in particular in the directory
/pub/usenet/news.answers/audio-fmts of the archive site
`rtfm.mit.edu`.

Octave simply treats audio data as vectors of samples (non-mono data are
not supported yet). It is assumed that audio files using linear
encoding have one of the extensions lin or raw, and that
files holding data in mu-law encoding end in au, mu, or
snd.

— Function File: **lin2mu** (x, n)  
> Converts audio data from linear to mu-law. Mu-law values use 8-bit
> unsigned integers. Linear values use n-bit signed integers or
> floating point values in the range -1<=x<=1 if n is 0.
> If n is not specified it defaults to 0, 8 or 16 depending on
> the range values in x.
>
>
>
> **See also:** mu2lin, loadaudio, saveaudio, playaudio, setaudio, record.

— Function File: **mu2lin** (x, bps)  
> Converts audio data from linear to mu-law. Mu-law values are 8-bit
> unsigned integers. Linear values use n-bit signed integers
> or floating point values in the range -1<=y<=1 if n is 0. If
> n is not specified it defaults to 8.
>
>
>
> **See also:** lin2mu, loadaudio, saveaudio, playaudio, setaudio, record.

— Function File: **loadaudio** (name, ext, bps)  
> Loads audio data from the file name.ext into the
> vector x.
>
> The extension ext determines how the data in the audio file is
> interpreted; the extensions lin (default) and raw
> correspond to linear, the extensions au, mu, or snd
> to mu-law encoding.
>
> The argument bps can be either 8 (default) or 16, and specifies
> the number of bits per sample used in the audio file.
>
>
>
> **See also:** lin2mu, mu2lin, saveaudio, playaudio, setaudio, record.

— Function File: **saveaudio** (name, x, ext, bps)  
> Saves a vector x of audio data to the file
> name.ext. The optional parameters ext and
> bps determine the encoding and the number of bits per sample used
> in the audio file (see `loadaudio`); defaults are lin and
> 8, respectively.
>
>
>
> **See also:** lin2mu, mu2lin, loadaudio, playaudio, setaudio, record.

The following functions for audio I/O require special A/D hardware and
operating system support. It is assumed that audio data in linear
encoding can be played and recorded by reading from and writing to
/dev/dsp, and that similarly /dev/audio is used for mu-law
encoding. These file names are system-dependent. Improvements so that
these functions will work without modification on a wide variety of
hardware are welcome.

— Function File: **playaudio** (name, ext)  
— Function File: **playaudio** (x)  
> Plays the audio file name.ext or the audio data
> stored in the vector x.
>
>
>
> **See also:** lin2mu, mu2lin, loadaudio, saveaudio, setaudio, record.

— Function File: **record** (sec, sampling\_rate)  
> Records sec seconds of audio input into the vector x. The
> default value for sampling\_rate is 8000 samples per second, or
> 8kHz. The program waits until the user types <RET> and then
> immediately starts to record.
>
>
>
> **See also:** lin2mu, mu2lin, loadaudio, saveaudio, playaudio, setaudio.

— Function File: **setaudio** ([w\_type [, value]])  
> Execute the shell command mixer [w\_type [, value]]

— Function File: y = **wavread** (filename)  
> Load the RIFF/WAVE sound file filename, and return the samples
> in vector y. If the file contains multichannel data, then
> y is a matrix with the channels represented as columns.
> — Function File: [y, Fs, bits] = **wavread** (filename)  
> > Additionally return the sample rate (fs) in Hz and the number of bits
> > per sample (bits).
> > — Function File: [...] = **wavread** (filename, n)  
> > > Read only the first n samples from each channel.
> > > — Function File: [...] = **wavread** (filename,[n1 n2])  
> > > > Read only samples n1 through n2 from each channel.
> > > > — Function File: [samples, channels] = **wavread** (filename, "size")  
> > > > > Return the number of samples (n) and channels (ch)
> > > > > instead of the audio data.
> > > > >
> > > > >
> > > > >
> > > > > **See also:** wavwrite.

— Function File: **wavwrite** (y, filename)  
— Function File: **wavwrite** (y, fs, filename)  
— Function File: **wavwrite** (y, fs, bits, filename)  
> Write y to the canonical RIFF/WAVE sound file filename
> with sample rate fs and bits per sample bits. The
> default sample rate is 8000 Hz with 16-bits per sample. Each column
> of the data represents a separate channel.
>
>
>
> **See also:** wavread.
